# Concerning the Etiology of Appendicitis1Read at the thirtieth annual meeting of the Alumni Association of the University of Buffalo, May 30, 1905.

**Published:** 1905-07

**Authors:** E. L. Shurly

**Affiliations:** Detroit


### BUFFALO MEDICAL JOURNAL.

VOL. XLIV.—LX. JULY, 1905. NO. 12

### ORIGINAL COMMUNICATIONS.

### Concerning the Etiology of Appendicitis.[1]

BY E. L. SHURLY, M. D., Detroit.

IN VIEW of the great amount of literature extant upon the subject of appendicitis, I would scarcely have the temerity to present a paper on any phase of the topic to any body of scientific men who were not so near and dear as fellow alumni. It is therefore only in the atmosphere of the family,—so to speak,—that I dare breathe forth suggestions on this world widely thrashed topic. I shall not surprise you by offering anything new, nor will I awe you with an abstract of so many hundred operations. I will not go so far as that. I will merely present to your notice a few suggestions regarding what is probably not known of its etiology, and what no fellow seems inclined to find out. I shall endeavor to give you as few platitudes as possible for a basis upon which to plant these humble suggestions.

To quote Morris:

The cecal appendage is vermiform in man and in all of the man like apes, also in certain Lemurs. In man, the cecal appendage is apparently a rudimental structure which once formed an important part of the alimentary tract. It is supposed that this organ, like the wisdom tooth, has degenerated during the process of evolution, as would any other unused structure. The appendix vermiformis in man was recognised as a structure in the 16th century and was described in the 18th century. (Lammoriere, however, believes this to be untrue). It appears at about the tenth week of fetal life. As you know, the length of an average appendix vermiformis in the young adult is not far from three and three quarter inches, with a diameter of a goose quill.

1. Read at the thirtieth annual meeting of the Alumni Association of the University of Buffalo, May 30, 1905.

It varies, however, in length from two inches to six inches, or a little more.

The contents of the appendix usually consists of mucus with more or less fecal matter and microörganisms. Under ordinary circumstances semisolid fecal matter and gas, find easy entrance to, and exit from, an appendix with a large lumen, as the appendix has abundant muscular ability to empty itself, and has at the seat of its attachment to the cecum a good fixed point for muscular action. In some of them the lumen is small, either naturally or from a little hyperplasia, and it is then, obviously, not so easy for the appendix to empty itself of its concretions. There are very many normal appendices containing concretions which cannot escape for this reason. Appendix concretions are of three principal sorts—fecal, phosphatic, and fatty. Insoluble salts may be precipitated out of the fermenting mucus, and as the stagnant mucus is very apt to undergo decomposition, the fecal concretions are usually arranged in layers with calcium salts.

Phosphatic concretions are formed in normal appendices, and in chronically affected appendices, as the result of decomposition of mucus.

The formation of fecal and phosphatic concretions, while more apt to occur in persons whose intestinal contents easily ferment, may be independent of any disease of the appendix, while fatty concretions probably occur as the result of long ulceration of the lymphoid coat only. Morris says, bacteria are by all means the most important things found in the appendix. Colon bacilli which have their normal habitat in the colon are almost invariably present in the lumen of the appendix. They are harmless dwellers there unless an infection atrium gives them an opportunity to migrate into the tissues. The pyogenic streptococci are also pretty constant dwellers in the normal appendix. Many of the less important pyogenic bacteria and saprophytes, or bacteria of fermentation harmlessly lurk in the appendix. When an infection atrium is made, the infection is at first mixed in character. The streptococci are apt to outstrip other bacteria in the second part of the race, and the colon bacilli are apt to lead finally. Thriving colonies of bacteria are daily swept along through the normal colon and are moved in and out of most appendices. Some of the higher entozoa are frequently found in the appendix. The nematode oxyuris, for instance, is often found there.

Various kinds of seeds are closely resembled by concretions. Morris and other surgeons state that they have not yet found a seed in any of the appendices which they have operated upon.

The appendix is the seat of fecal concretions in at least 8 per cent. of all cases. Their existence does not denote that the appendix is diseased!

Morris's definition of appendicitis is, that it is an infectious exudative inflammation of the appendix vermiformis ceci, originating in any local cause for the production of an infected atrium in the tissues of the appendix, and progressing by bacterial invasion into the layers of connective tissue, and into the layers of lymphoid tissue—all of which are partially or completely disabled by interstitial exudate compression within the narrow muscular peritoneal sheath of the appendix. The principal cause of appendicitis is mixed bacterial infections from the lumen of the appendix. The chief cause of bacterial infection from the lumen of the appendix is the formation of an infection atrium in the mucosa of the appendix by force applied in any way. He further says, I formerly surmised that the appendix was sometimes injured by pressure between a full cecum and a hard pelvic wall—supposing that the cecum was often filled with fecal matter. But after extensive opportunities and observation, I have not as yet seen fecal matter in the cecum at any operation, and there is doubt if the so-called impaction is not often lymph exudate instead. Excepting in elderly people, I believe that injury to the mucosa occurs most often from accidental twisting of the appendix upon part of its long axis. An infection atrium is also commonly produced by erosion of the mucosa at the site of a concretion, or by entozoa. Bacterial infection may extend into the tissues of the appendix from the infected cecum, as in typhoid fever or dysentery. An infection atrium is formed consequent upon peritonitis, extending from adherent infected oviducts or other nearby structures.

Dr. J. B. Murphy, as you all know, has written some fine papers on this subject. The etiology he gives is about as follows:

When the disease was first recognised as a surgical affection, the acute infective lesions were considered to be due to the usual causes which produced enteritis in the remaining portion of the intestine and it was considered essentially a disease of the summer months. Now, since we have become more familiar with it, we find that it is almost as common in winter as in the summer. We find that it does not come on under the same climatic or dietary conditions as the other types of enteritis; that it most commonly follows exposures such as would produce socalled "cold" of the respiratory tract. So commonly is it a sequence of such exposure that a very prominent Chicago physician at one time considered it essentially a rheumatic manifestation. This, however, we do not accept.

Foreign bodies, as fruit seeds, gallstones, capsules, and the like, were present in a little less than 2 per cent. of the cases coming under observation; fecal concretions were found in 38 per cent. and we believe that the erosion of the appendiceal mucosa by these foreign bodies produces an atrium for the admission of the infective flora into the tissues and precipitates under favorable conditions the acute attack. Indiscretions in diet appear to have little if any effect as an etiological factor.

The types of infective flora found in cultures and stainings from the appendices vary in the following order of frequency: bacillus coli communis, staphylococcus pyogenes aureus and albus; streptococcus, bacillus tuberculosis, actinomycoses.

Appendicitis occurs in all classes with about equal frequency. In my work it has been a little more frequent in males than in females. I do not consider that it is contagious or infectious. It does seem, however, to have family predilections. This I would rather consider due to the conformation of the appendix or the diminished resistance to infection in some families and the comparative immunity to infection in others. I have not infrequently had two members of the same family in bed with the disease at the same time.

Many surgeons believe in an anatomical cause for this disease, maintaining that appendices which are spiral, club-shaped, too long, with too much lymphoid tissue, or bent in their formation, or adherent, will act for the supervention of the disease. Some writers adduce as evidence, the advent of attacks of appendicitis in families where abnormalities or peculiar anatomical types are supposed to occur through hereditary succession. Still other writers seek to account for the occurrence of the disease in females by assuming previous disease or malformation of the uterus or its appendages.

In a report of Peterson on 200 cases of chronic disease of the uterine appendages, 106 or 58.4 per cent. were accompanied by normal appendices, while 41.6 per cent. showed past or present changes in the organ. In this same report, Peterson says that the appendix was adherent in 18.5 per cent. Fecal concretions were noticed twelve times out of 146 observations or 8 per cent. The shape of the appendix was noted as being abnormal in 52 out of the 200 cases or 26 per cent. Out of the 200 cases, 106 or 53 per cent. showed practically no evidence of disease!

Peterson also states that in chronic diseases of the uterine appendages adhesions of the accompanying appendices are present in nearly 50 per cent. of the cases where microscopic examination shows the latter to be diseased. In a certain proportion of cases, however, although the appendix may be adherent it is also perfectly normal. In chronic diseases of the uterine appendages the appendix which is club-shaped, constricted or contains fecal concretions, is not necessarily diseased. In 30 per cent. of patients with uterine fibromata there is accompanying disease of the appendix; also in 70.9 per cent. of patients with ovarian cystomata.

He and other writers state that only a little over 50 per cent. of appendices removed during the course of operation on pelvic lesions will be found microscopically to be normal. The remainder will show forms of acute and chronic inflammation or the result of former inflammation. The appendix is adherent twice as often in those cases where microscopic examination shows past or present disease. A certain proportion of adherent appendices are, however, perfectly normal microscopically. Mere shape of the appendix can not serve as an index of its normality or disease. Appendices may be club-shaped, constricted or bent upon themselves, and yet be perfectly normal microscopically.

Championniere believes that appendicitis is comparatively a new disease and that its advent is in some way related with la grippe. He gives various statistics to show that its prevalence is coincident or sequent upon endemics of la grippe or influenza. Besides this, he calls attention to facts which he supports by statistics to show that the majority of cases occur among the meat eaters; that in countries in Europe where the people subsist mostly upon vegetable food, cases of appendicitis are rare. Many writers believe that the contributory cause is constipation or subacute disease of the intestines; such as, enteritis or dysentery. Among them Sherrill believes that constipation and resulting malnutrition are important causes.

The concensus of opinion, however, seems to be that the important cause is the accession and activity of various bacteria, and specifically the colon bacillus through abrasion of the lining membrane; but to assign to these bacteria any more than a secondary or exciting role in the causation of the disease would seem irrational, when we come to reflect that there are millions of colon bacilli in the intestines of every individual, and even on the face and hands and in the mouth all the time, and that there are large numbers of staphylococcus, streptococcus and various saprophytes (which may become pathogenic) continually on and about one's person, and the appurtenances with which we come in contact.

Concerning the formerly supposed causation of appendicitis—by the presence of concretions and various foreign bodies—modern experience has brought to light, as indicated by the quotations which we have already made, that many appendices harbor such things as well as the bacteria without becoming necessarily diseased. Modern research has also demonstrated that all sorts of anatomical variations in the form of the appendix has been observed without the organ thereby becoming diseased, and that previous diseases of the uterine appendages are not invariably the causes.

In explanation of all this the theory is advanced that if through mechanical action, or otherwise, an abrasion or atrium is formed by which these microörganisms—especially the colon bacillus (which is always dwelling in this little pocket)—secure an entrance into the connective tissue and lymph vessels, they thereby set up irritation, then infection, and so on; that in this habitat—which has been liken to a culture tube—they are constantly setting up fermentation, forming toxins which are ready to gain access through some accidental abrasion, to the lymph or capillary vessels with the usual result of producing infection, and the like; and, further that concretions, etc., are probably the principal agents in producing such abrasions by mechanical action.

Although this undoubtedly is the process which actually takes place after the initial step—namely, the abrasion—has been made, the questions still remain whether in all cases an abrasion constitutes the first step? And what previous condition must exist to provide an atrium or abrasion? For we have seen, according to the present state of our knowledge, that anatomical abnormalities exist, concretions and other foreign bodies are tolerated. Various pathogenic bacteria including the colon bacillus are, probably, always present—being shown in over 50 per cent. at least of appendices examined and found healthy; to say nothing of the thousands of appendices which are never brought to light, and which are never diseased throughout life, and which it is fair to presume are in like circumstances. Besides the evidence first advanced, a general survey of human pathology and human physiology would seem to suggest an insupperable objection to making this little accident (the formation of an atrium) the pivot around which the pathogenesis of the disease in all cases must revolve. This would hardly account for the large number of cases among healthy people of all ages, from infancy to senility, which are met with. There must therefore be something beyond, or before, which induces the accession of the disease. It seems to me, therefore, that more enterprise is needed in studying the domestic history of cases of appendicitis in order to work out antecedent causes.

For instance: what are the details of life which the infant or child has been subjected to, who is attacked with appendicitis? What influence has the diet, the exercise or play had? What injuries, if any? What abnormalities, if any? What characterises the life of this infant or child? With adults, what has been the antecedent habits, mental and physical, of the patient? What particular occupation? Whether any previous diseases of the intestines or stomach has existed? What particular sort of diet—whether vegetable or animal? Has an irregular fermentation of the food or drink waged an influence? What, if any, effect has the alcoholic habit or social condition of the patient had? Or, in other words, do persons addicted to the moderate or excessive use of alcohol become liable or not to appendicitis? Do persons consuming principally a vegetable or an animal diet render themselves liable to appendicitis? Do persons who take their food without proper mastication thus become liable? Do persons of sedentery occupations thus become liable? Do persons of active occupation, such as the laborer or mechanic, thus become more or less liable? Do persons living an out-door or an in-door life thus become more or less liable? Are persons of certain nationality more liable than others? Is the white man particularly liable, or the negro and Malay less liable? Has hereditary dyscrasia or diathesis anything to do with it?

These are suggestions which I am sure have entered the mind of every one of you present today. Yet how few surgeons and physicians have tried to trace up the etiology of appendicitis along any of these lines? In the town which I live, I think it can safely be stated that, very few cases of appendicitis occur among the laboring class, which is composed largely of Poles, Hungarians, Italians and negroes. If so, why? There is much investigation needed, the evidence so far on this point being far from clear, because the data recorded are too insignificant to lead to a definite conclusion.

Concerning constipation which many surgeons believe to be the principal predisposing cause, we shall find a small percentage of constipated persons who are attacked with appendicitis. In my small range of observation I have been very much puzzled to observe that many persons who are in the habit of taking more or less mineral water or saline cathartics, or even vegetable cathartics, have been attacked with appendicitis. Among these many will be found who are great eaters; and strange as it may seem, but a very small percentage of alcoholic drinkers seem to become subjects of appendicitis. This observation has also amazed me, and I have been wondering whether the alcohol has anything to do with either changing or obstructing certain fermentations, or whether its irritant effect upon the peripheral nerve ends of the intestines stimulated peristalsis to such an extent as to prevent the occurrence of the disease by changing the contents of the appendix oftener. All of these questions and many others naturally crowd upon us when we think over the large number of cases which are occurring and the total inadequacy of the preliminary causes as laid down to account for them.

I would, therefore, in conclusion suggest that a thorough investigation into the antecedent daily life of every patient afflicted with appendicitis be made—very much in the same manner that an attorney with a given case proceeds to trace up circumstantial evidence. I would suggest that this evidence might include the details of occupation, and the details concerning the eating and drinking of the individual; as to the kind of food and the amount; the kind of drink, be it water or anything else, and the quantity of such drink that has been ingested for some time previous to the attack. In short, a thorough investigation into all the domestic vicissitudes relating to the patient, in order to ascertain what causes concretions, and the like, of the appendix, and how comes the abrasion.

In this way it seems to me that we might be enabled to discover what prediposes to make this little pouch a culture tube, what deflects the natural course of fermentation into one of infectious fermentation, and what the necessary conditions are for such a deflection of natural processes. As we cannot open the abdomen of every individual who comes to us and ascertain by our vision just what is going on, it seems to me that this method, although more tedious, should be adopted in the hope that we may arrive at the anterior causes and by our advice direct the people toward such physiological methods of living as would prevent the development of this prevalent and serious disease. There is but one other course before us in the way of prevention, until the time shall come when this rudimentary appendix shall finally disappear as an element of human intestinal anatomy,—and that is, to extend to the realm of surgery the task of opening every child's abdomen and removing the appendix soon after birth. This idea, however, is too utopian for the present, unless perhaps we could get such a procedure adopted as part of a religious code —in which case undoubtedly appendicitis might the sooner be stamped out.

32 Adams Avenue, West.

---

Mortality Among American Physicians.—During 1902 the deaths of 1,400 regular practitioners of the United States were noticed in the *Journal of the American Medical Association,* which number is probably 5 per cent. of the total mortality in the profession of this country. The number of regular practitioners in the United States is about 95,000, making the death rate for the year about 14.74 per thousand, a lower rate than is usually supposed to obtain. The mean average age at death was 58 years, 7 months and 29 days, and the average previous duration of practice was 28 years, 10 months and 28 days.—*Denver Med. Times.*

